# Interspecies Regulation Between *Staphylococcus caprae* and *Staphylococcus aureus* Colonized on Healed Skin After Injury

**DOI:** 10.3389/fmicb.2022.818398

**Published:** 2022-03-01

**Authors:** Kohei Ogura, Hiroka Furuya, Natsuki Takahashi, Kana Shibata, Maho Endo, Shinya Watanabe, Longzhu Cui, Tohru Miyoshi-Akiyama, Shigefumi Okamoto, Kazuhiro Ogai, Junko Sugama

**Affiliations:** ^1^Advanced Health Care Science Research Unit, Institute for Frontier Science Initiative, Kanazawa University, Kanazawa, Japan; ^2^Department of Clinical Laboratory Science, Faculty of Health Sciences, Institute of Medical, Pharmaceutical and Health Sciences, Kanazawa University, Kanazawa, Japan; ^3^Division of Bacteriology, Department of Infection and Immunity, Faculty of Medicine, Jichi Medical University, Shimotsuke, Japan; ^4^Pathogenic Microbe Laboratory, Research Institute, National Center for Global Health and Medicine, Shinjuku, Japan; ^5^AI Hospital/Macro Signal Dynamics Research and Development Center, Institute of Medical, Pharmaceutical and Health Sciences, Kanazawa University, Kanazawa, Japan; ^6^Research Center for Implementation Nursing Science Initiative, School of Health Sciences, Fujita Health University, Toyoake, Japan

**Keywords:** pressure injury, healed skin, recurrent injury, skin microbiome, single locus sequence typing, *Staphylococcus aureus*, *Staphylococcus caprae*

## Abstract

*Staphylococcus* spp. colonize commensally on the human skin. Some commensal coagulase-negative staphylococci and *Staphylococcus aureus* are also involved in nosocomial infections. Bacteria were collected from skin healed from pressure injury (PI). After the collection time points, some patients suffered from recurrent PI (RPI). This study analyzed the characteristics of *Staphylococcus* spp. on healed skin before recurrence between healed skin that suffered from RPI within 6 weeks (RPI group) and healed skin that did not suffer within the duration (non-RPI group) by *Staphylococcus* spp.-specific sequencing. Of the seven patients in the RPI group, two were dominated by *S. aureus* and four by *Staphylococcus caprae*, coagulase-negative human commensal staphylococci in the RPI group. Using mouse models, both *S. caprae* and *S. aureus*, but not *Staphylococcus epidermidis*, colonized on skin healed from injury at significantly higher rates than normal skin. Although subcutaneous injection of *S. caprae* did not induce lesion formation, the bacterium exhibited high hemolytic activity on human red blood cells. Lesion formation by subcutaneous injection of *S. aureus* was significantly suppressed in the presence of *S. caprae*. The hemolytic activity of rabbit blood cells of *S. aureus* was suppressed by *S. caprae*, whereas the hemolytic activity of *S. caprae* was dramatically suppressed by *S. aureus*. Data indicated that each of the two *Staphylococcus* spp. suppresses the pathogenicity of the other and that the imbalance between the two is associated with RPI.

## Introduction

The skin is covered with numerous microorganisms, mainly bacteria (Kong, 2011). Bacteria reside on the epidermis and form commensal microbiota (Byrd et al., 2018). The skin microbiome is affected by skin microbiotopes, such as sebaceous, moist, and dry environments, and host factors, such as age and gender (Grice and Segre, 2011; Oh et al., 2014; Shibagaki et al., 2017). Although the skin’s microbial composition is not usually changed for a duration of years (Oh et al., 2016; Ogai et al., 2021a), in case of damage to the skin’s barrier function, the microbiome has an imbalance, followed by an increase of pathogenic bacteria, leading to cutaneous inflammatory disorders (Belkaid and Segre, 2014; Naik et al., 2015). For example, recent studies have shown that the skin microbiome is associated with diabetic foot ulcer, one of the major types of chronic wounds (Kalan and Brennan, 2019). On the diabetic foot ulcer, *Staphylococcus* spp. and *Corynebacterium* spp. tend to be highly prevalent, followed by mixed anaerobic communities (Loesche et al., 2017). Grice et al. (2010) found in mouse models that a longitudinal shift in the diabetic wound microbiota is correlated with prolonged skin defense response.

During aging, resident immune cells such as macrophages (Langerhans cells) and dendritic epidermal T cells become easily damaged, affecting the normal organization of the skin and its capacity for repair. Numerous mechanisms are involved in age-induced skin degradation; they also relate to non-healing or chronic wounds in the elderly (Farage et al., 2013; Blume-Peytavi et al., 2016; Russell-Goldman and Murphy, 2020). Pressure injuries (PIs) or pressure ulcers are clinical problems in bedridden people and develop by prolonged mechanical loading on the skin over a bony prominence, mostly at the sacral area (Schank, 2015). The mean incidence of PIs in Japanese long-term care hospitals is low (1.9%; Igarashi et al., 2013). In contrast to the low frequency of primary PI incidence, recurrent PIs (RPIs) are much higher, from 25.9% (Arisandi et al., 2020) to 26.7% (Shibata et al., 2021), in long-term care hospitals, although preventive care is constantly provided. Therefore, besides the occurrence of PIs, RPIs are deemed another clinical problem in a rapidly growing aging society.

In order to find out new factors associated with RPI, we have been focusing on skin microbiome. In a previous study, 16S rRNA-based sequencing of the healed site microbiome revealed significantly lower alpha diversity (Shannon index) and higher rates of *Staphylococcus* spp. in the RPI group than in the non-RPI group (Shibata et al., 2021). Since *Staphylococcus* spp. contain *S. aureus*, the dominant bacterial skin pathogen, we had suspected dominance of this bacterium on the healed sites which subsequently suffered from RPI. However, quantitative polymerase chain reaction (qPCR) showed that *S. aureus* did not show a significant difference between the groups, suggesting the involvement of other coagulase-negative staphylococci (CoNS). It is well known that CoNS could be involved in skin protections. For example, *S. epidermidis* and *S. hominis* produce antimicrobial peptides (AMPs) that synergize with the human cathelicidin AMP LL-37 and suppress colonization of *S. aureus* on human skins with atopic dermatitis (Nakatsuji et al., 2017). *S. lugdunensis* also inhibits *S. aureus* growth by producing AMP called lugdunin (Zipperer et al., 2016). In addition, recent reports showed that CoNS primes the skin immune system to limit the colonization of potential invaders (Parlet et al., 2019). In contrast, CoNS also could cause pathogenicity (Hatlen and Miller, 2021). Some *S*. *epidermidis* strains produce EspA proteases, which involve skin damages, while the amounts of the production vary among the strains (Cau et al., 2021). While *S. lugdunensis* inhibits *S. aureus* growth as mentioned above (Zipperer et al., 2016), this bacterium also possesses invasive pathogenic potential (Heilbronner and Foster, 2021).

These reports suggest that inter-species regulation of *Staphylococcus* spp. and mechanisms of pathogenicity are complicated. In order to analyze and clarify the relationships, identification of *Staphylococcus* spp. on the patients’ skins was required. Although 16S rRNA metataxonomics targeting the V3–V4 regions has been widely used for the determination of microbial populations (Wang et al., 2007; Claesson et al., 2010; Willis et al., 2019), this method lacks a species-level resolution; thus, the study could not identify which *Staphylococcus* spp. are related to RPIs. To determine *Staphylococcus* spp. associated with RPIs, this study conducted a sequencing method called single-locus sequence typing (SLST), recently developed by Scholz et al. for the strain-level identification of *Propionibacterium acnes* (Scholz et al., 2014; Scholz and Jensen, 2017) and utilized for *Staphylococcus* profiling on the skin (Smits et al., 2020). Based on the SLST profiling results of the healed site microbiome, *Staphylococcus caprae*, a coagulase-negative staphylococcus, and *S. aureus* reside on healed PIs. Although *S. caprae* has been regarded as a commensal bacterium in goat milk, the bacterium has been isolated from a patient with invasive infectious disease (Watanabe et al., 2018). Although it is known that hemolysin production of *S. aureus* is suppressed by AIP produced by goat milk-isolated *S. caprae* (Paharik et al., 2017), the pathogenicity of human-isolated *S. caprae* and cross-talk between the *S. caprae* and *S. aureus* have not been not fully clarified. This study presented the association of these two *Staphylococcus* spp. and the implications of these bacteria in the deterioration of skin integrity.

## Materials and Methods

### Study Design

In this study, first we aimed to identify *Staphylococcus* spp. colonized on the skins healed from a PI. In order to analyze the pathogenicity of human-isolated *S. caprae* and cross-talk between the *S. caprae* and *S. aureus*, next we conducted some experiments such as mouse infection models and *in vitro* hemolysis assay.

### Clinical Samples and Participants

A prospective cohort study was conducted in a long-term care hospital with 500 beds in Ishikawa Prefecture (Japan) from 2018 to 2019. Skin samples were collected from 30 patients who had healed from a PI within 1 month, although one sample was excluded because it was obtained immediately after bathing. After sample collection, whether the RPI (lesion or redness) was present at the sites where the samples were collected was assessed every week. Within 6 weeks, seven from a total of 29 patients suffered from RPI (Shibata et al., 2021). Written informed consent was obtained from the participants or her/his guardians before inclusion in the study. Patients who had a skin disease, hypertrophic scars, keloids, or other PIs were excluded. Further information on the participants was described in a previous report (Shibata et al., 2021).

### PCR and Sequencing

DNA samples from the 29 patients were obtained by attaching an air-permeable medical tape with urethane glue to a healed PI site or a control site, according to a previous report (Ogai et al., 2021b). The control site was opposite the healed PI site when the healed PI was present on a trochanter or ∼5 cm superior to the healed PI site when present on the sacrum or coccyx (Arisandi et al., 2020). Sequence data of 16S rRNA genes (V3–V4 regions) were obtained in a previous report [DNA Data Bank of Japan (DDBJ) accession no. DRA009678 (Shibata et al., 2021)]. To determine *Staphylococcus* spp. associated with RPI, this study conducted SLST, a recently developed sequencing method that can type and profile the targeted bacterial family beyond the species level (Ederveen et al., 2019). This study selected a *Staphylococcus*-specific variable region in a gene encoding 30S ribosomal protein S11, which was chosen by a TaxPhlAn pipeline using 7,247 *Staphylococcus* genomes, for an SLST target. The regions were amplified by KAPA HiFi HS ReadyMix (Roche, Basel, Swiss) and a pair of SLST PCR primers (Supplementary Table 1), followed by purification by AMPure XP magnetic beads (Beckman Coulter, Brea, CA, United States). After the addition of index sequences using the Nextera XT Index Kit version 2 (Illumina, San Diego, CA, United States) and subsequent purification by AMPure, the equimolar mixtures of the products were applied for MiSeq sequencing with the MiSeq Reagent Kit version 3 (Illumina) and 15% of PhiX Control version 3 (Illumina). Sequence data were deposited to the DDBJ (accession no. DRA013063).

### Single-Locus Sequence Typing Analysis

The 300-bp pair-end reads (fastq data) were filtered (QC > 20) and merged by the fastp tool (Chen et al., 2018), followed by conversion into FASTA-format sequence data by the seqtk tool.^1^ Sequence data were applied to the TaxPhlAn pipeline (Ederveen et al., 2019). The alleles were built with a Shannon diversity index threshold of 0.6. Unassigned alleles were manually extracted and applied to nucleotide BLAST in the National Center for Biotechnology Information website and regarded as those derived from species with the highest scores.

### Assembly of 16S rRNA Metataxonomics and Single-Locus Sequence Typing Data

A 16S rRNA metataxonomic analysis was previously conducted for identical samples, and the abundance of *Staphylococcus* spp. in the microbiota was determined (Shibata et al., 2021). 16S rRNA and SLST data were assembled by adjusting the abundance of each *Staphylococcus* spp., which were obtained by SLST, to the abundance of *Staphylococcus* spp. in the whole microbiota obtained by 16S rRNA metataxonomics (Supplementary Figure 2).

### Bacterial Strains

*Staphylococcus caprae* ATCC 35538 [strain designation: 143.22 (CCM 3573)] (Devriese et al., 1983), derived from goat milk, and *Staphylococcus epidermidis* ATCC 14990 [strain designation: Fussel (NCTC 11047, R. Hugh 2466)] were obtained from the American Type Culture Collection. *S. caprae* JMUB145 was isolated from the blood culture of a woman with fever during chemotherapy treatment for uterine cancer at Jichi Medical University Hospital in Japan (Watanabe et al., 2018). *S. aureus* N315 (*agr* type II) was kindly provided by Dr. Keiichi Hiramatsu (Kuroda et al., 2001).

### Genomic DNA of *Staphylococcus caprae*

After incubation in TE buffer containing lysostaphin and lysozyme at 37°C for 30 min, genomic DNA was extracted by the QIAamp DNA mini kit (Qiagen, Hilden, Germany) according to the manufacturer’s instructions.

### Quantitative Polymerase Chain Reaction

To analyze the allele types of the *agrD* gene in the clinical samples, qPCR was conducted using QuantiFast Probe PCR kits (Qiagen), primers, and probes described (Supplementary Table 1) in the AriaMx Real-time PCR System (Agilent Technologies, Santa Clara, CA, United States). Before the analysis, amplification specificity was validated using the indicated copy numbers of ATCC35538 and JMUB145 genomes that possess the *agrD*_YSTCSYYF and *agrD*_YRTCNTYF genes, respectively. To prepare standards, genomic DNA was extracted using QIAamp DNA mini kit according to the manufacturer’s instruction. Double-stranded DNA (dsDNA) concentrations were measured using the Qubit dsDNA HS Assay Kit (Thermo Fisher Scientific, Waltham, MA, United States).

### Colonization of Staphylococci on Mouse Backs

Five-week-old female BALB/c mice (Sankyo Laboratory Service Corporation, Tokyo, Japan) were anesthetized through intraperitoneal administration of a mixture of Dormicum (4 mg/kg; Astellas Pharma, Tokyo, Japan), Vetorphale (5 mg/kg; Meiji Seika Pharma Co., Ltd., Tokyo, Japan), and Domitor (0.3 mg/kg; Nippon Zenyaku Kogyo Co., Ltd., Fukushima, Japan), followed by disinfection of mouse backs by ethanol. A circular full-thickness wound (4 mm in diameter), including the panniculus carnosus muscle, was made using a sterile biopsy punch (Kai Industries Co., Ltd., Gifu, Japan). Seven days after wound formation, the backs of control (no surgery) and healed mice were shaved under anesthesia by the Dormicum/Vetorphale/Domitor mixture. A 10 μL aliquot containing no bacteria [negative control for counting colony-forming units (CFUs) of commensal bacteria], 2.0 × 10^7^ CFUs of *S. aureus* N315, 3.2 × 10^7^ CFUs of *S. caprae* JMUB145, or 3.5 × 10^7^ CFUs of *S. epidermidis* ATCC 14990 [strain designation: Fussel (NCTC 11047, R. Hugh 2466)] in phosphate-buffered saline (PBS) was inoculated directly onto the scabs or control sites by a micropipette. The droplets were confirmed to be dried under anesthesia. On Day 2 post-infection, mice were euthanized with sevoflurane (Maruishi Pharmaceutical Co., Ltd., Japan). The skin (∼1 × 1 cm) was collected by sterile scissors, suspended in sterile PBS, and mixed vigorously by a vortex mixer.

### Subcutaneous Injection

Eight-week-old female BALB/c mice were anesthetized through intraperitoneal administration of the Dormicum/Vetorphale/Domitor mixture. Mouse backs were shaved and disinfected by ethanol. A 100-μl bacterial solution in PBS was injected to the subcutis in two separate experiments in triplicate for each group. The *S. aureus* strain N315 (1.3 × 10^8^–1.9 × 10^8^ CFU/mouse), *S. caprae* strain JMUB145 (1.3 × 10^8^–1.9 × 10^8^ CFU/mouse), *S. caprae* strain ATCC 35538 (1.1 × 10^8^–1.5 × 10^8^ CFU/mouse), *S. epidermidis* ATCC strain 14490 (1.9 × 10^8^–2.7 × 10^8^ CFU/mouse), and coinjection of *S. aureus* strain N315 and *S. caprae* strain JMUB145 were tested. The size and color intensity of lesions were calculated by ImageJ. To quantify the intensity, image files (pictures) were converted to grayscale, the lesions were selected by drawing/selection tools, and the area-integrated intensity was calculated.

Using normal skin with the same size (1 cm apart from the selected area) as the control, the intensity was calculated as (intensity of the lesion)/(intensity of the normal skin).

### Hemolysis Assay

*Staphylococcus aureus* N315, *S. caprae* JMUB145, and *S. caprae* ATCC 35538 were cultured in tryptic soy broth (TSB; Becton Dickinson, United State) overnight at 37°C. After centrifugation at 6,000 × *g* at 10 min, spent medium (supernatant) was collected. Cultured bacteria were suspended in fresh TSB or TSB containing spent medium (final 10%) at a density of OD_600nm_ = 0.001. After overnight culture and centrifugation at 6,000 × *g* at 10 min, spent media were utilized for analysis.

Human blood was obtained from 22 to 38-year-old healthy volunteers. Human whole blood was collected in EDTA-containing tubes and centrifuged at 1,500 × *g* at 20 min. After washing with PBS thrice, cells were suspended at 20% (v/v) in PBS containing 0.5% bovine serum albumin (BSA; FujiFilm Wako Pure Chemical Corporation, Osaka, Japan). Rabbit whole blood in Alserver’s solution (Kohjin-Bio, Saitama, Japan) was centrifuged at 1,200 × *g* at 20 min. Rabbit blood cells were washed with PBS containing 0.1% BSA four times and suspended at 20% (v/v) in PBS containing 0.5% BSA.

The human or rabbit red blood cell (RBC) solution (200 μL) was incubated with 20 μL spent medium or fresh TSB (for blank control) for 6 h (human) or 18 h (rabbit). After centrifugation at 1,500 × *g* at 20 min, the absorbance of the supernatants was measured at a wavelength of 492 nm.

### Growth Curve

The spent medium was obtained after overnight culture, centrifuged at 6,000 × *g* at 10 min, and filtered. *S. aureus* N315 and *S. caprae* JMUB145 were suspended in fresh TSB or TSB containing spent medium (final 10%) at a density of OD_600nm_ = 0.001 and cultured at 37°C with shaking for 10 h.

### Ethics Statement

The clinical study (Kanazawa University no. 44) and animal experiments (Kanazawa University no. AP-214236) were approved by the Medical Ethics Committee of Kanazawa University and conducted in accordance with the Declaration of Helsinki and the Microorganism Safety Management Regulations of Kanazawa University.

## Results

### Assembly of Staphylococci-Specific Single-Locus Sequence Typing and 16S rRNA Sequence Data

**FIGURE 1:**
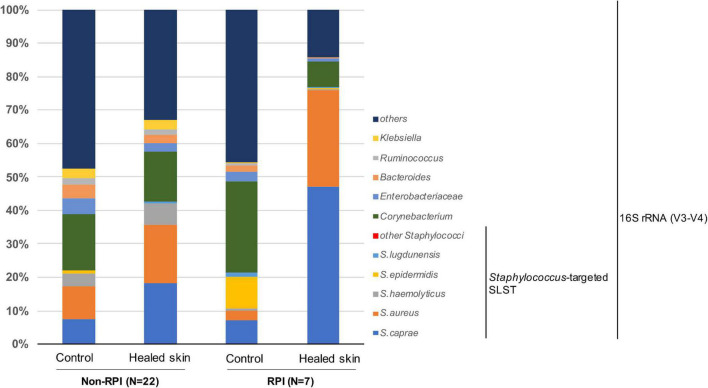
Composition of *Staphylococcus* spp. and the top five skin bacteria on the skin sites. Average abundance was shown for the control site of non-RPI patients (*n* = 22), healed skin sites of non-RPI patients (*n* = 22), control sites of RPI patients (*n* = 7), and healed skin sites of RPI patients (*n* = 7). The full sets of staphylococci are shown in Supplementary Figure 2. The composition of each sample is shown in Supplementary Figure 3.

**FIGURE 2:**
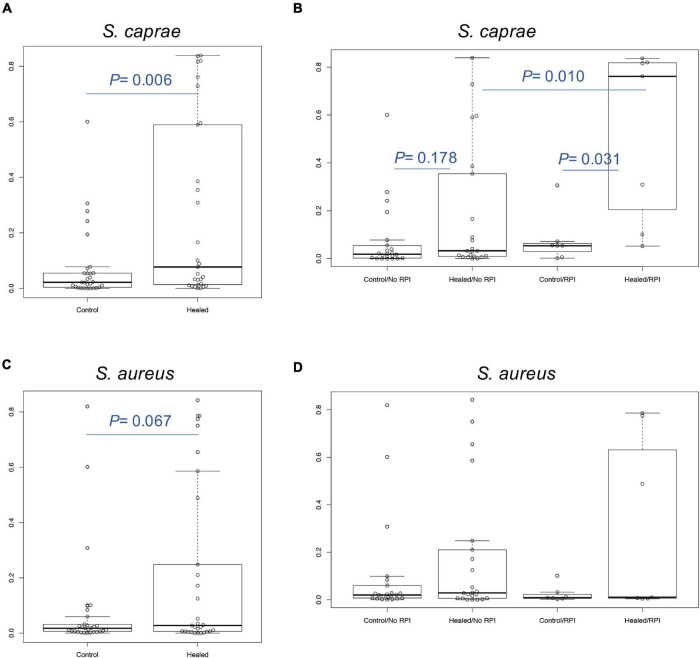
Relative abundance of *S. caprae* and *S. aureus*. Comparison of **(A)** *S. caprae* and **(C)** *S. aureus* abundance between control sites (including non-RPI and RPI) and healed sites. *P*-values were calculated by Welch’s *t*-test. **(B)** Comparison of *S. caprae* and the four groups. After the Kruskal–Wallis test (*p* = 0.006), Bonferroni correction was calculated for statistical significance. **(D)** Comparison of *S. aureus* and the four groups. No significance was detected by the Kruskal–Wallis test.

We focused on skin microbiome associated with RPI. In a previous report, 16S rRNA metataxonomics revealed that the abundance of *Staphylococcus* spp. was significantly higher in RPI-healed sites than non-RPI-healed sites (*p* = 0.002, Mann–Whitney *U* test) (Shibata et al., 2021) and that the abundance was also significantly in the healed sites (including RPI and non-RPI) than the control sites (*p* < 0.01, Welch’s *t*-test), indicating composition of staphylococci is involved in RPI. In this study, *Staphylococcus* spp. were identified using a recently developed sequencing method named SLST. A *Staphylococcus*-specific region in a gene encoding 30S ribosomal protein S11 was amplified by PCR and sequenced by the MiSeq system. After merging pair-end reads, removing low-quality reads, and converting read data to multi-FASTA format, data were applied to the TaxPhlAn pipeline (Ederveen et al., 2019). The pipeline gave numbers of sequences derived from each *Staphylococcus*. The abundance of each *Staphylococcus* was adjusted to that of *Staphylococcus* spp. obtained by 16S rRNA sequencing in the previous report (Figure 1 and Supplementary Figures 2, 3). *Staphylococcus* spp. were mainly dominated by *S. caprae* and *S. aureus*, whereas the abundance of *S. epidermidis* was low. The abundance of *S. caprae* was significantly higher in healed sites than control sites (*p* < 0.01, Welch’s *t*-test; Figure 2A). The abundance of *S. caprae* was also significantly higher RPI-healed sites than non-RPI-healed sites (*p* = 0.01, Kruskal–Wallis test and Bonferroni correction; Figure 2B). *S. aureus* was high in healed sites (*p* = 0.07, Welch’s *t*-test; Figure 2C). The multiple comparison procedure (Kruskal–Wallis test) did not detect significance among the four groups (Figure 2D). There was no significant difference in the abundance s of *Staphylococcus haemolyticus* or *S. epidermidis* (Supplementary Figure 4). As shown in our previous report, there was no significant difference in the abundance of *Corynebacterium* spp. between RPI-healed sites and non-RPI-healed sites (Shibata et al., 2021) but RPI-control sites and RPI-healed sites (*p* = 0.02, Kruskal–Wallis test and Bonferroni correction) (Supplementary Figure 4).

### *Staphylococcus caprae* and *Staphylococcus aureus* in the Recurrent Pressure Injury Group

**FIGURE 3:**
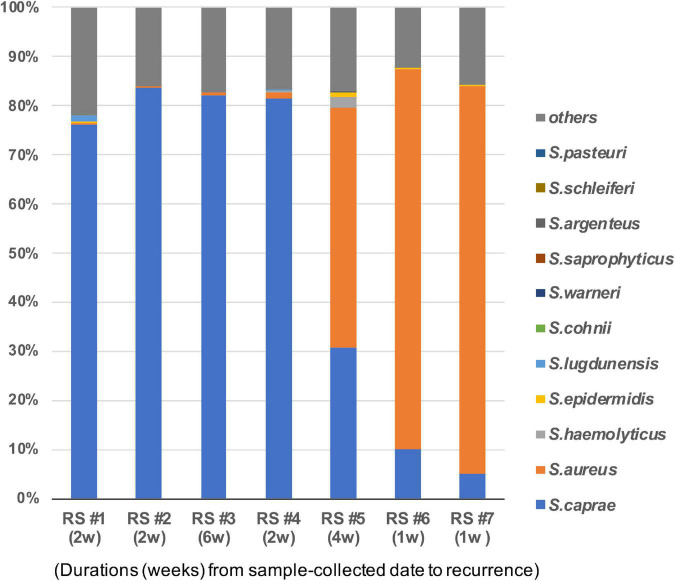
*Staphylococcus caprae* and *S. aureus* on healed skin of the seven patients who suffered from RPI within 1–6 weeks. Bacterial composition of the seven sites. Duration from the sample collection date to RPI is described in parentheses. The correlation of the abundance of *S. aureus* and *S. caprae* is shown in Supplementary Figure 4.

This study observed the *Staphylococcus* composition of the seven healed skins that suffered recurrence 1–6 weeks later. The dominance of *S. caprae* was found in four of the seven patients, whereas that of *S. aureus* was found in the other two samples (RS #6 and #7 in Figure 3). In one patient’s skin (RS #5) that suffered recurrence in 4 weeks, both *S. caprae* and *S. aureus* colonized. Patients who had the *S. aureus*-dominant microbiome suffered from RPI within 1 week, whereas those with the *S. caprae*-dominant one did after 2–6 weeks. There were no significant characteristics in the patient information.

### *Staphylococcus caprae* Strains

**FIGURE 4:**
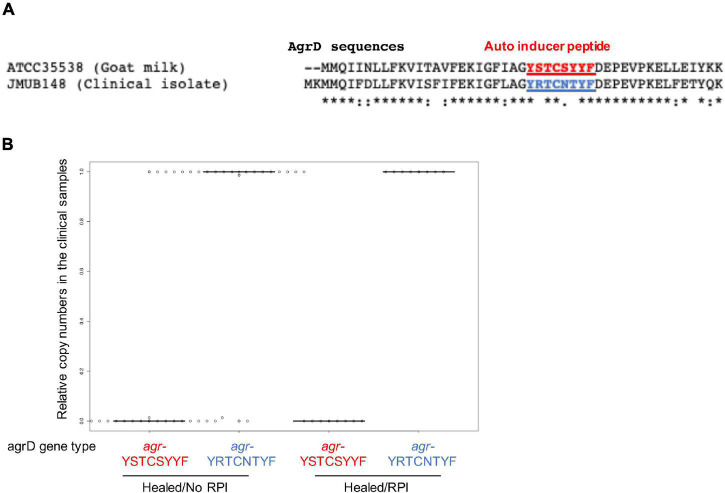
*Staphylococcus caprae* typing by *AgrD* genes in the clinical samples. **(A)** Different amino acid sequences of AgrD from ATCC 35538 (goat milk) and JMUB145 (clinical isolate), although mature structures of the peptides have not been confirmed yet. **(B)** Copy numbers were calculated by probe qPCR using the serial dilution of purified chromosomal DNAs of ATCC 35538 (*agrD*_YSTCSYYF) and JMUB145 (*agrD*_YRTCNTYF). Relative copy numbers were calculated as (*agrD*_YSTCSYYF or *agrD*_YRTCNTYF copy numbers)/[(*agrD*_YSTCSYYF copy numbers) + (*agrD*_YRTCNTYF copy numbers)].

*Staphylococcus caprae*, CoNS, usually colonize goat skin and milk and occasionally cause goat mastitis (Devriese et al., 1983). Recent reports showed that *S. caprae* also reside as human commensal bacteria and occasionally cause nosocomial infections (Seng et al., 2014). Two *S. caprae* strains ATCC 35538 and JMUB145, isolated from goat and human, respectively, showed 98.5% average nucleotide identity (Yoon et al., 2017), indicating that their characteristics were slightly different. Some of staphylococci such as *S. aureus* is typed by an accessory-gene-regulator (*agr*) gene sequence (Dufour et al., 2002). Paharik et al. (2017) reported that *S. caprae* from goat milk possesses an *agr* gene encoding autoinducing peptide YSTCSYYF. Watanabe et al. (2018) reported that clinical isolate *S. caprae* possesses another allele type of the *agrD* gene encoding YRTCNTYF (Figure 4A), although mature structures of the peptides have not been confirmed yet. To determine which type of *S. caprae* was found in the healed ulcers, qPCR was conducted to count the copy numbers of *agrD*_YSTCSYYF and *agrD_*YRTCNTYF genes (Supplementary Figure 6). Most *agrD* genes in the samples were *agrD*_YRTCNTYF in healed sites (Figure 4B), indicating that genotypes of *S. caprae* strains in the clinical samples are similar to *S. caprae* JMUB145 rather than ATCC 35538. Thereafter, this study mainly utilized the human isolate *S. caprae* JMUB145 for analysis.

### Colonization on Mouse Scab

**FIGURE 5:**
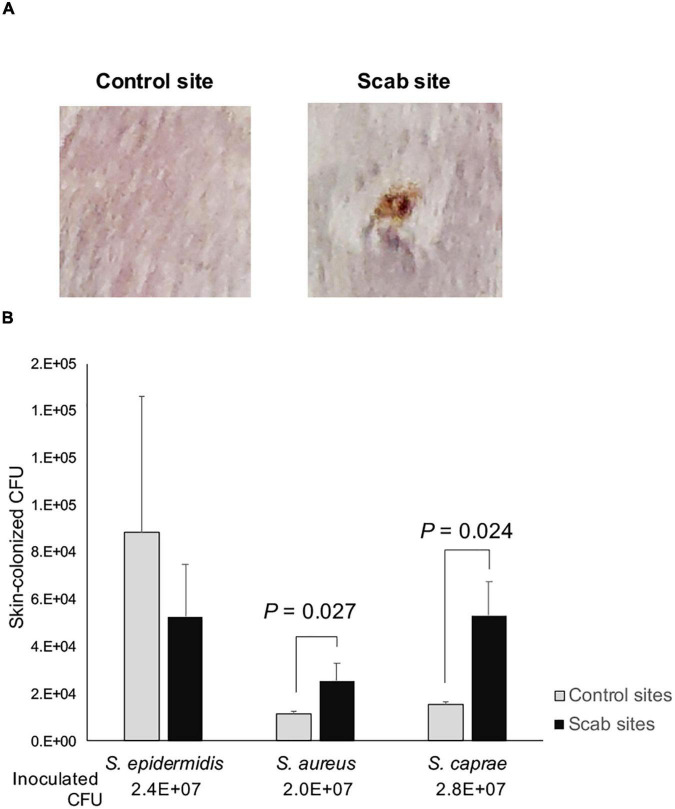
Skin colonization of staphylococci. **(A)** Representative images of control and healed skin. To prepare skin healed from wounds, mouse skin was punched out, and wounds were left for 1 week. **(B)** Colonized bacteria. On Day 2 post-infection, skin (1 × 1 cm) was suspended in PBS and vigorously mixed by vortexing. The relative CFU values were calculated as (CFUs of colonized bacteria)/(CFUs of infected bacteria). *p*-Values were calculated by Student’s *t*-test.

This study indicated that *S. caprae* and *S. aureus* colonized on the healed PI site. To examine the ability of colonization on the skin after wound healing, a full-thickness wound on the back of mice was made, and 7 days were allowed to pass for the wound to heal (Figure 5A). Using this skin scab model, PBS containing *S. caprae*, *S. aureus*, and *S. epidermidis* were spotted (inoculated) onto the control (i.e., uninjured) and scab sites. The colonized bacteria were collected and counted on Day 2 postinoculation. *S. epidermidis* (strain ATCC 14990) colonized on the control skin at a significantly higher ratio than skin scab (*p* < 0.05, one-way analysis of variance) than *S. aureus* (*agr* type II strain N315) and *S. caprae* (strain JMUB145), although the ratio was not altered when this bacterium was inoculated on skin scab (Figure 5B). In contrast, *S. aureus* N315 and *S. caprae* JMUB145 colonized on skin scab at a significantly higher ratio than control skin (*p* < 0.05, Student’s *t*-test), suggesting that both *S. aureus* and *S. caprae* prefer colonization on healed skin rather than on the normal skin.

### Subcutaneous Injection

**FIGURE 6:**
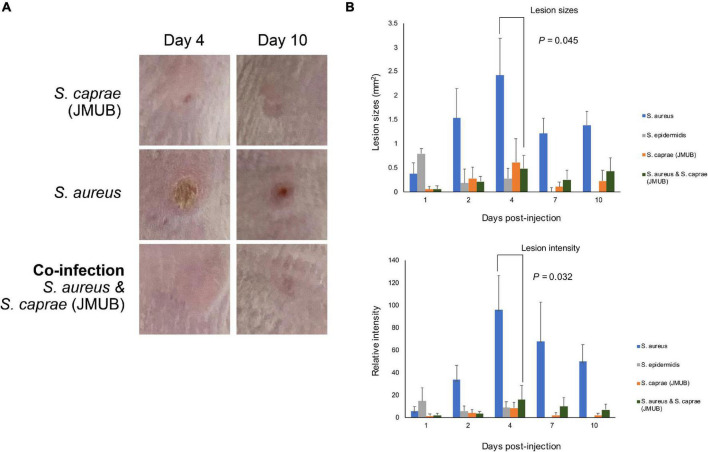
Subcutaneous injection. **(A)** Representative images of lesions after subcutaneous injection. Picture sizes are 1 × 1 cm. The other images are shown in Supplementary Figure 5. **(B)** Lesion sizes and intensity. The color intensity was calculated by ImageJ using normal skin (1 cm apart from injured sites) as control (=1). *p*-Values were calculated by Student’s *t*-test.

The mouse model revealed that both *S. caprae* and *S. aureus* could colonize on healed skin (Figure 5). However, it remained unknown whether the dominance of these bacteria is directly associated with the deterioration of skin integrity, such as RPIs. In the colonization experiments (Figure 5), there was no significant inflammation or ulcer recurrence at the bacteria-inoculated healed skin, suggesting that another model is required to be developed to resemble RPI. Although several reports have shown how to mimic PI in animal models, it is still very difficult to mimic the RPI situation with animal models. Thus, this study analyzed the pathogenicity of *S. caprae* and *S. aureus* by a subcutaneous injection model. Subcutaneous injection of *S. aureus* strain N315 resulted in lesion formation with the largest size and highest color intensity on Day 4 (Figure 6 and Supplementary Figure 7). In contrast, injection of *S. caprae* JMUB145 alone or *S. caprae* ATCC 35538 alone did not result in lesion formation. When *S. aureus* N315 and *S. caprae* JMUB145 were co-injected, the resultant lesion sizes were significantly smaller than those caused by *S. aureus* alone (Figure 6).

### Suppression of Rabbit Blood Hemolysis of *Staphylococcus aureus* by *Staphylococcus caprae*

**FIGURE 7:**
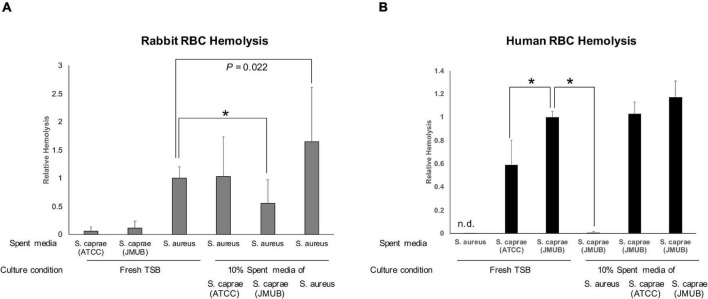
Hemolysis by spent medium. Autoinducer peptides are indicated by underlines. **(A)** Lysis of rabbit blood cells. Fresh or spent medium of *S. aureus* N315, *S. caprae* ATCC 35538, or *S. caprae* JMUB145 was added to the final 10%, followed by incubation of *S. aureus* N315 overnight. *S. caprae* ATCC 35538 and *S. caprae* JMUB145 were incubated in fresh medium. Spent media were utilized for the assay. **(B)** Human blood cells. Fresh or spent medium of *S. aureus* N315, *S. caprae* ATCC 35538, or *S. caprae* JMUB145 was added to the final 10%, followed by incubation of *S. caprae* JMUB145 overnight. *S. aureus* N315 and *S. caprae* ATCC 35538 were cultured in fresh medium. *P*-values were calculated by Student’s *t*-test. An asterisk indicates *p* < 0.001.

α-Hemolysin (Hla) plays a key virulence factor in the pathogenicity of *S. aureus* (Bayer et al., 1997). Regarding the suppression of Hla production, Paharik et al. (2017) reported that an autoinducer peptide composed of eight amino acids (YSTCSYYF) with a C-terminal five-membered thiolactone ring produced by *S. caprae* from goat milk could suppress Hla production and lesion formation of *S. aureus* on mouse skin. Their result was consistent with this study. Watanabe et al. (2018) reported the complete genome sequences of three *S. caprae* strains isolated from patients with nosocomial infections in Japan. Inconsistent with the report by Paharik et al. (2017) the three *S. caprae* strains isolated in Japan, including strain JMUB145, possessed *agrD* genes that encode different eight amino acids (YRTCNTYF; Figure 4A). To examine whether the two *S. caprae* agrD peptides (YSTCSYYF and YRTCNTYF) exhibit different characteristics to Hla production by *S. aureus*, this study tested the effects of AgrD peptides from *S. caprae* strains ATCC 35538 (*agrD*_YSTCSYYF) and JMUB145 (*agrD*_YRTCNTYF) on the hemolysis of rabbit RBCs by *S. aureus*. Spent media obtained from overnight TSB culture was utilized for hemolytic assay. Bacteria were also cultured in the presence of 10% spent medium. Hemolytic activity was tested using spent medium. As shown in Figure 7A, *S. aureus* N315 exhibited hemolytic activity on rabbit RBCs. The hemolytic activity was significantly enhanced when *S. aureus* N315 was cultured in the presence of 10% *S. aureus* N315 spent medium (*P* = 0.003, Student’s *t*-test). In contrast, the hemolytic activity using spent medium was partially suppressed by culture in the presence of 10% *S. caprae* JMUB145 (*p* < 0.001) but not ATCC 35538 (*p* = 0.898), indicating that ability of suppressing Hla production by *S. aureus* is different between the two *S. caprae* strains. In the previous report by Paharik et al. (2017) half maximal effective concentration (EC_50_) was shown, indicating that concentration might be required to examine the inhibitory effects of the ATCC 35538 supernatants.

### Suppression of Human Blood Hemolysis of *Staphylococcus caprae* by *Staphylococcus aureus*

Hla exhibited hemolytic activity on rabbit RBCs but not human RBCs (Klainer et al., 1972). Consistently, the spent medium of *S. aureus* N315 did not exhibit hemolytic activity on human RBCs (Figure 7B). In contrast, *S. caprae* strains ATCC 35538 and JMUB145 possess several genes encoding β-hemolysin, δ-hemolysin, and putative hemolysins (Watanabe et al., 2018). The two *S. caprae* strains exhibited hemolytic activity on human RBCs (Figure 7B). Interestingly, the activity of *S. caprae* strain JMUB145 was significantly higher than the *S. caprae* strain ATCC 35538 (*p* < 0.001, Student’s *t*-test). Moreover, hemolysis was dramatically suppressed by culture in the presence of 10% *S. aureus* N315 spent medium (*p* < 0.001), showing that hemolysin production by *S. caprae* is suppressed by *S. aureus*. The spent medium did not affect the growth speed of *S. aureus* or *S. caprae* (Supplementary Figure 8).

## Discussion

Wound formation changes the skin microenvironment consisting of commensal bacteria and significantly changes the skin microbial community (Johnson et al., 2018). *Staphylococcus* spp. dominates chronic wounds (e.g., diabetic foot ulcers) (Wolcott et al., 2016; Gardiner et al., 2017). Gardner et al. (2013) revealed that the relative abundance of *Staphylococcus* spp. in chronic wounds is associated with wound duration. In this study, the microbiome of the skin healed from PI was analyzed and the composition of commensal *Staphylococcus* spp. was compared. The analysis showed the dominance of *S. caprae* on healed skins that suffered from RPI (four of the seven patients) within 1–6 weeks after healing (Supplementary Figure 1). Contrastingly, the relative abundance of *S. epidermidis* was extremely low in all skin sites (Figure 1). Therefore, *S. caprae* might have an inhibitory effect on *S. aureus* and/or *S. epidermidis* in the skin microenvironment during the healing process of RPI. Some CoNS species compete with *S. aureus* on specific skin microenvironments. *S. epidermidis* produces an AMP to eliminate *S. aureus* (Nakatsuji et al., 2017) and a phenol-soluble modulin to promote immune defense in the wound (Cogen et al., 2010). *S. capitis* was predominantly found in skin samples derived from patients with lesional atopic dermatitis compared with *S. aureus* and *S. caprae* (Smits et al., 2020). Similar to other CoNS, *S. caprae* might play an important role in the recovery process of skin damage from RPI.

It is well known that there is a crosstalk between *S. aureus* and CoNS *via* the *agr* quorum sensing system (Canovas et al., 2016), which contains *agr*ACDB genes and a regulatory RNA (RNAIII) (Novick et al., 1995; Reynolds and Wigneshweraraj, 2011). The *agr* locus is ubiquitous in *S. aureus* and in CoNS. An autoinducing peptide (AIP), which is encoded and secreted by *agrDB* genes, binds to histidine kinase (AgrC), followed by promoting RNAIII expression *via* AgrC-dependent phosphorylation of the response regulator (AgrA) and upregulating specific virulence genes (Wang et al., 2014). In *S. aureus*, AIP accumulation leads to the upregulation of α-hemolysin (Queck et al., 2008), whereas this upregulation is suppressed by competitive binding of CoNS AIPs to *S. aureus* AgrC (Ji et al., 1997; Olson et al., 2014). AIPs have genetic and functional diversity, and this variety makes the intra/interspecies crosstalk *via* the *agr* systems complicated because sensitivity and specificity of AIP significantly vary depending on the combination of *agr* types. The *agr* of *S. aureus* strains is classified into four groups (I–IV) (Ji et al., 1997; Otto et al., 2001; Geisinger et al., 2012). It was also reported that the *agr* of other CoNS also has a sequence variety (Olson et al., 2014). Otto et al. (2001) reported that *S. epidermidis* AIP type I specifically inhibits *S. aureus agr* type III quorum sensing but not the other types. Regarding *S. caprae*, although AIP produced by a goat-isolated *S. caprae* strain inhibits all the four *agr* sensing systems, the effects were less toward type IV than the other three types (Paharik et al., 2017). In this study, it was found that *S. caprae* that colonized on the patients carried *agr* genes encoding different AIP from the goat-isolated one (Figure 4). Though all reported staphylococcal AIPs contained a five-membered thiolactone ring with an N-terminal extension (Thoendel and Horswill, 2013; Olson et al., 2014), the mature structure of the human-isolated *S. caprae* AIP remains unknown. Additionally, there are a few limitations in the present *in vivo* and *in vitro* studies (Figures 5–7). First, only the *S. aureus* N315 strain encoding a type II *agr* system was tested. Second, the suppression of α-toxins (Figure 7) might not be due to the *agr* system but another system such as *saeR/S* system in *S. aureus* (Voyich et al., 2009; Guerra et al., 2016). Thus, further analysis is necessary for the elucidation of *agr*-dependent crosstalk between human-isolated *S. caprae* and *S. aureus* such as testing other *agr* types of *S. aureus* strains, using synthetic AIPs of the human-isolated *S. caprae*, deleting the *agrDBC* genes, and/or exchanging the *agr* genes with those of the goat-isolated *S. caprae*.

As shown in Figure 3, the two patients with a high abundance of *S. aureus* on their healed ulcers suffered from RPI within 1 week, whereas the other four patients with a high abundance of *S. caprae* suffered from RPI between 1 and 6 weeks. These data showed that the balance of *S. aureus* and *S. caprae* could be related to RPIs; especially, the dominance of *S. aureus* can result in RPI at an earlier time point (i.e., within 1 week from healing). However, due to the small numbers of samples (only two samples of the *S. aureus*-dominant microbiome), it remained unclear whether such an imbalance of the two species is actually involved in RPI. *S. caprae* is considered part of a healthy human skin flora (Gowda et al., 2018). *S. caprae*, first isolated from goat milk, colonizes the skin and mammary glands of goats. This bacterium is also found in healthy human skin and can become pathogenic in humans (Seng et al., 2014; Mazur et al., 2017). The human-isolated *S. caprae* (strain JMUB145) displayed hemolytic activity on human RBCs (Figure 7A), indicating that this bacterium possesses pathogenicity to the skin of older people, although direct evidence was not obtained through the mouse models. Watanabe et al. (2018) reported that the three human-isolated *S. caprae* commonly possesses genes encoding phenol-soluble modulins (PSMs), including ∂-toxin, PSM-α, PSM-β, and PSM-δ, while the genes were also conserved in goat-isolated *S. caprae* strain ATCC 35538.

There were a few limitations for giving knowledge to clinical implementation through this study. Although this study attempted to investigate whether these *Staphylococcus* spp. are involved in RPI, a direct relationship between RPI and staphylococci remained unknown. It is challenging to develop RPI animal models. Although among the seven samples of the RPI groups, there was a significantly negative correlation between *S. caprae* and *S. aureus* (Figure 3 and Supplementary Figure 4), such negative correlation was on the basis of the relative abundance and did not directly elucidate the increase or decrease in amounts of colonized bacteria. This study could not count the absolute quantity using the present methods. Nevertheless, this study found new characteristics of human-isolated *S. caprae*, such as dominance on elderly skin healed from PI (Figure 2), the ability of colonization on mouse skin healed from injury (Figure 6), suppression of *S. aureus*-producing Hla, and production of hemolysin targeting human RBCs (Figure 7). In conclusion, this study showed that interspecies regulation between *S. caprae* and *S. aureus* on healed skin is associated with RPI.

## Data Availability Statement

The datasets presented in this study can be found in online repositories. The names of the repository/repositories and accession number(s) can be found in the article/Supplementary Material.

## Ethics Statement

The studies involving human participants were reviewed and approved by the Medical Ethics Committee of Kanazawa University. The patients/participants provided their written informed consent to participate in this study. The animal study was reviewed and approved by the Medical Ethics Committee of Kanazawa University.

## Author Contributions

KoO designed the study, analyzed all data, and wrote the manuscript. HF conducted the animal experiments and hemolysis assay. NT collected the clinical samples and analyzed their sequence data. KS contributed to the sample collection. ME contributed to the animal experiments. SW revised the discussion and improved quality of the manuscript. SW and LC contributed to the experiments using *S. caprae* strains. TM-A analyzed the genomic data of *S. caprae*. SO supervised HF and ME and contributed to the launch of this project and its application for animal experiments. KaO conceived this study, collected sequence data, and revised the manuscript critically for content. JS launched this project, supervised NT and KS, and funded this study. All authors contributed to the article and approved the submitted version.

## Conflict of Interest

The authors declare that the research was conducted in the absence of any commercial or financial relationships that could be construed as a potential conflict of interest.

## Publisher’s Note

All claims expressed in this article are solely those of the authors and do not necessarily represent those of their affiliated organizations, or those of the publisher, the editors and the reviewers. Any product that may be evaluated in this article, or claim that may be made by its manufacturer, is not guaranteed or endorsed by the publisher.

## Footnotes

1
https://github.com/lh3/seqtk

## Abbreviations

PI: pressure injury
RPI: recurrent pressure injury
SLST: single-locus sequence typing
*agr*: accessory-gene-regulator
RBC: rabbit red blood cell.
