# Supplementary material for: Interspecies Regulation Between Staphylococcus caprae and Staphylococcus aureus Colonized on Healed Skin After Injury
Source: Front Microbiol. 2022 Mar 1;13:818398. doi: 10.3389/fmicb.2022.818398 (PMC8921658; doi:10.3389/fmicb.2022.818398)
Supplement: Supplementary file 1 [file Data_Sheet_1.PDF]

Supplementary Material

Interspecies Regulation between *Staphylococcus caprae* and *Staphylococcus aureus* Colonized on Healed Skin after Injury

Kohei Ogura[1], Hiroka Furuya[2†], Natsuki Takahashi[1†], Kana Shibata[1], Maho Endo[1], Shinya Watanabe[3], Longzhu Cui[4], Tohru Miyoshi-Akiyama[5], Shigefumi Okamoto[1,2], Kazuhiro Ogai[6*], Junko Sugama[1,7*].

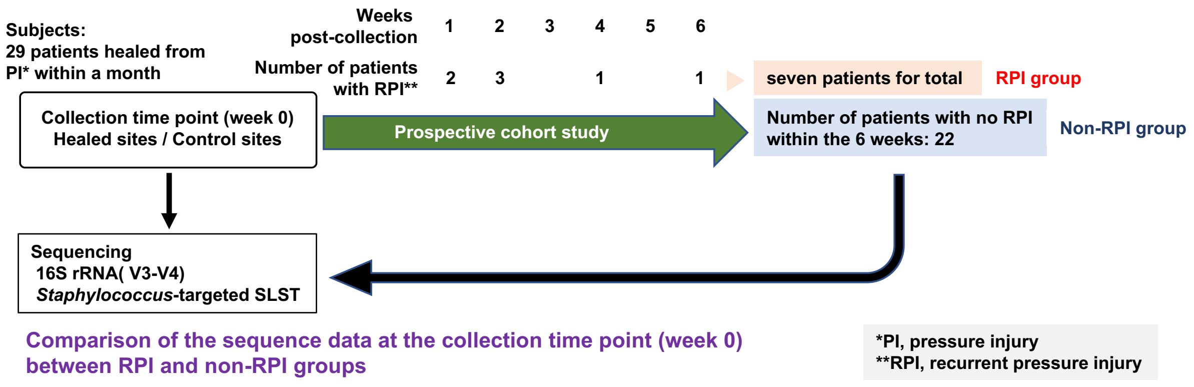

Figure S1. Study design of our clinical study.

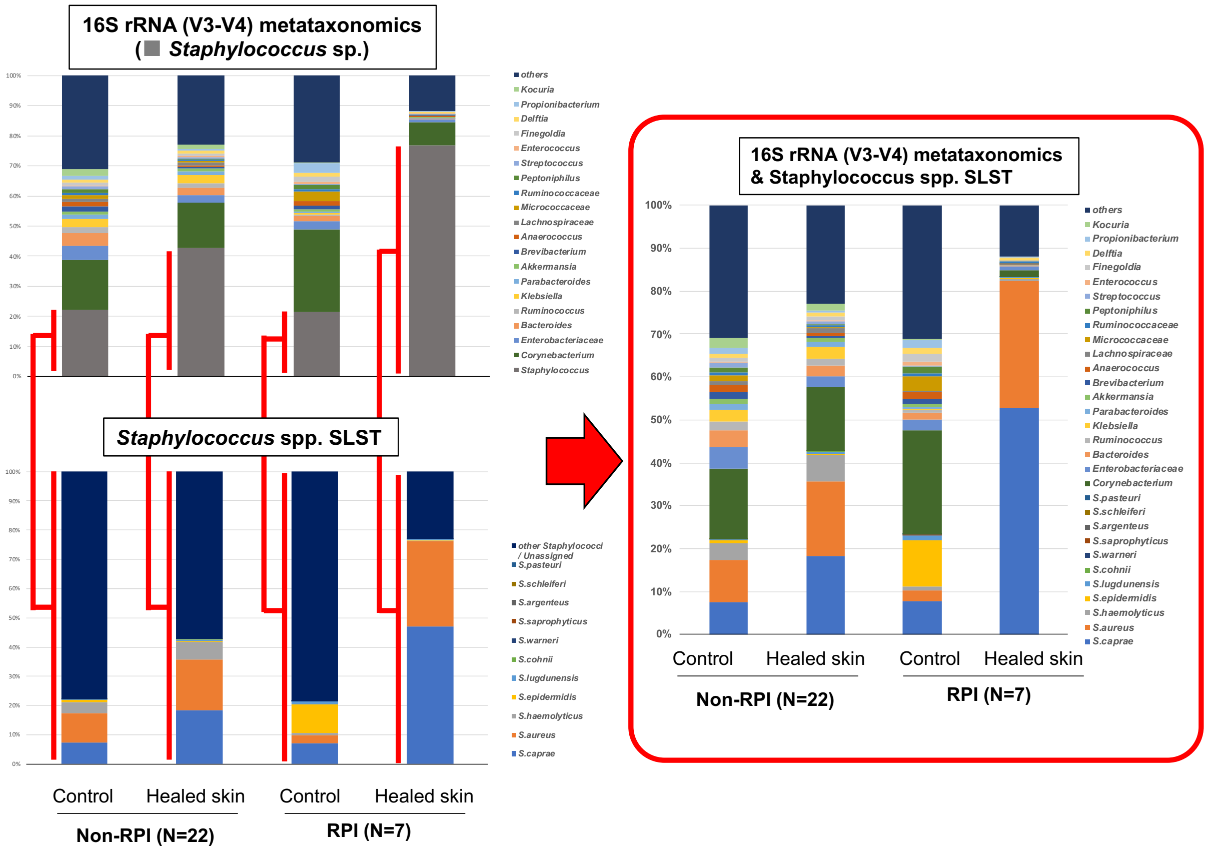

Figure S2. Combination of 16S rRNA metataxonomics and *Staphylococcus* spp. SLST (related to Figure 1). Average abundance was shown for the control site of non-RPI patients, healed skin of non-RPI patients, control site of RPI patients, and healed skin of RPI patients.

frontiers

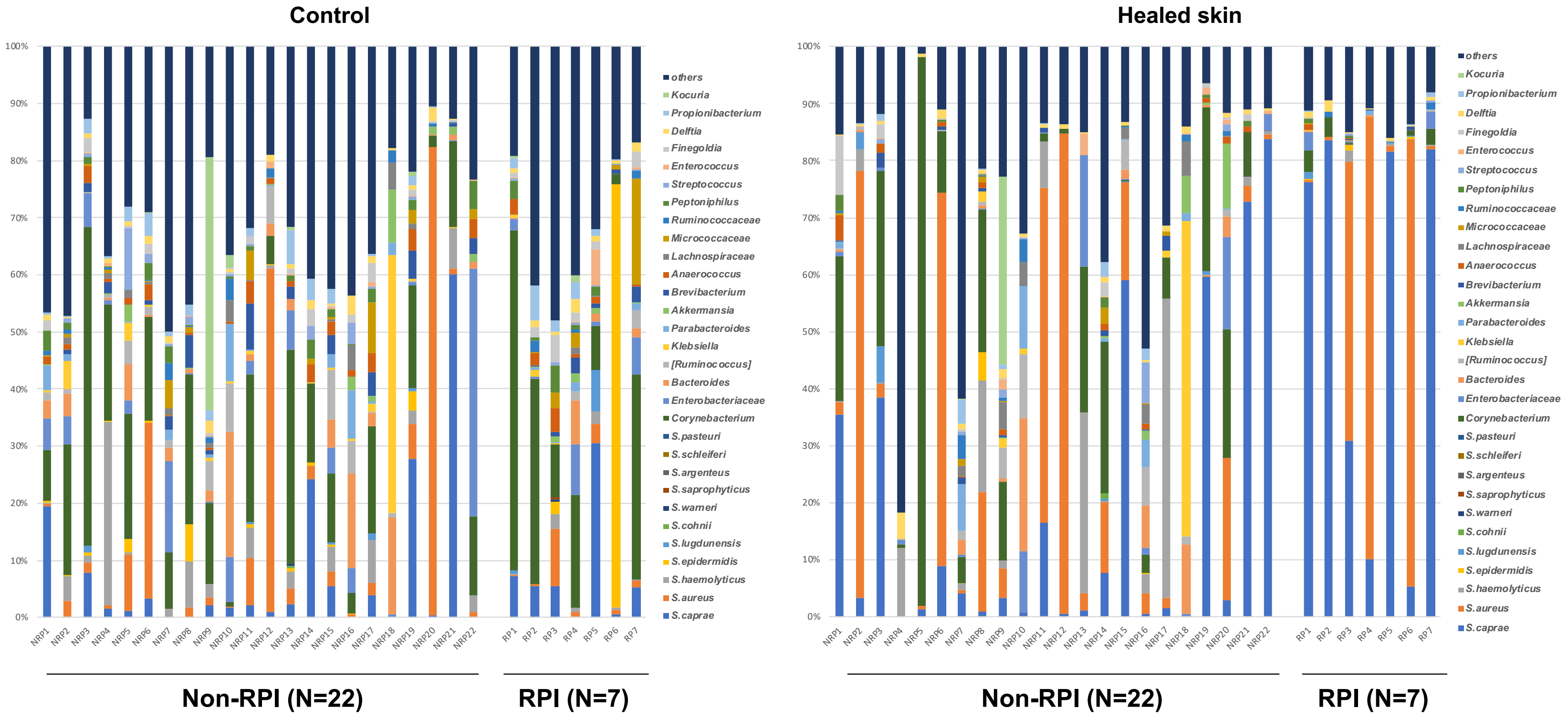

Figure S3. Microbiome abundance of each sample (Related to Figure 1).

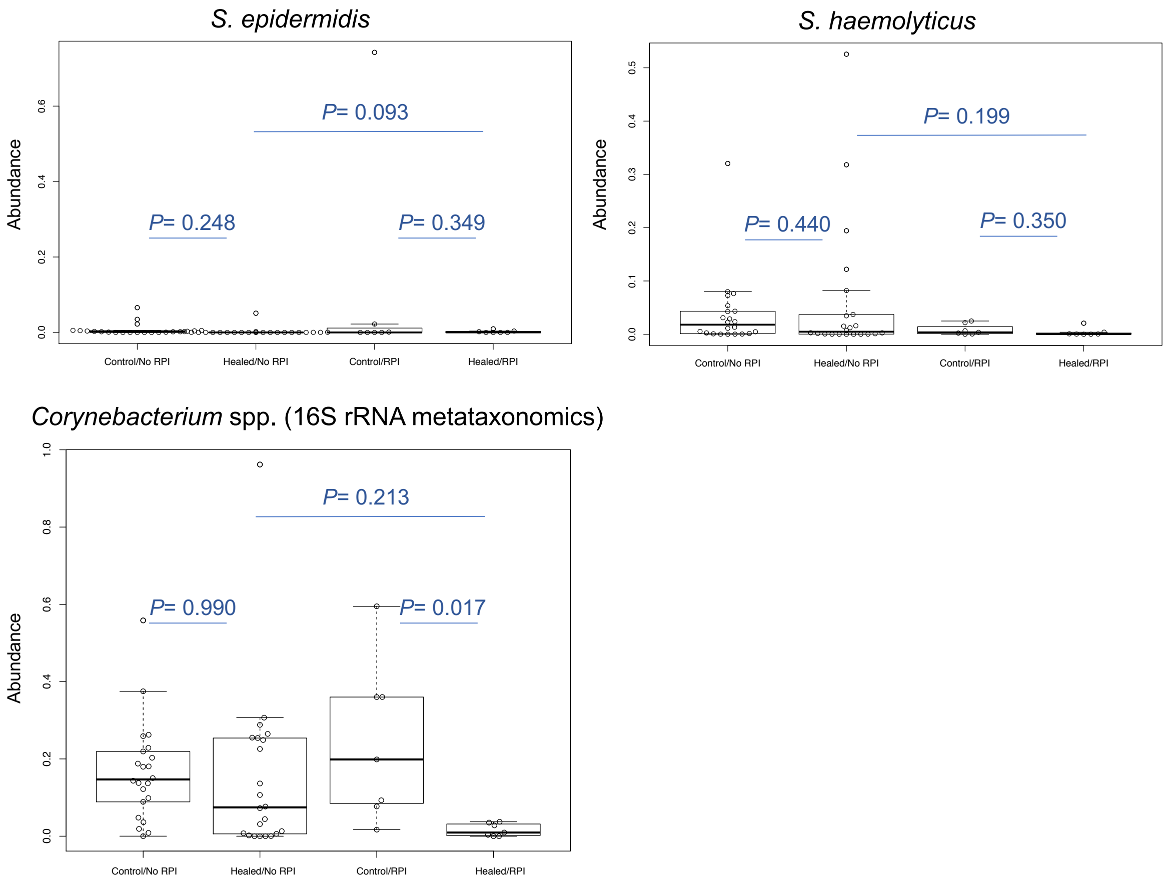

Figure S4. Relative abundance of *S. epidermidis*, *S. haemolyticus,* and *Corynebacterium* spp. (related to Figure 2). *P* values were calculated by Student's t-test between control and healed ulcers and the Mann–Whitney U test between non-RPI and RPI-healed ulcers. Abundance of *Corynebacterium* spp. was calculate using 16S rRNA metatoxonomics data.

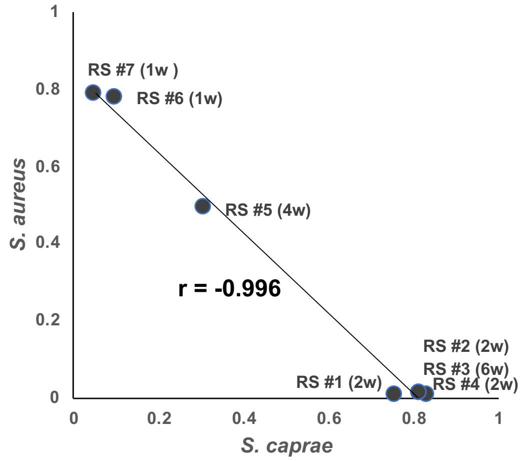

Figure S5. Correlation of abundance of *S. aureus* and *S. caprae* (related to Figure 3).

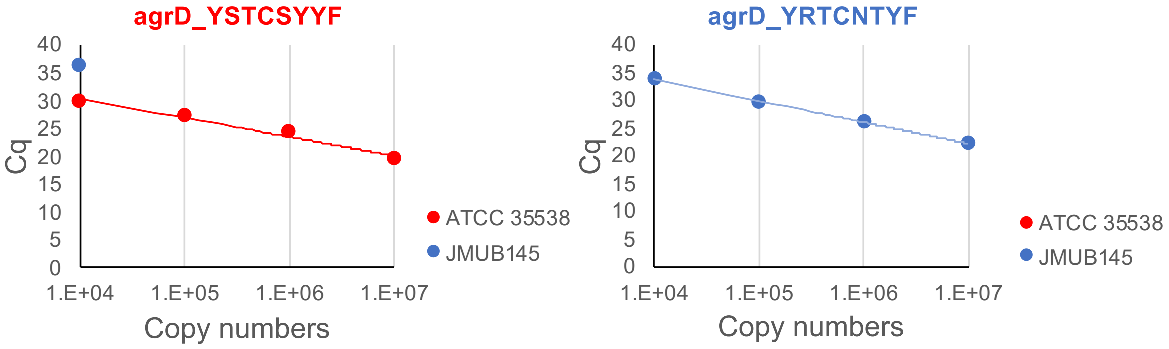

Figure S6. qPCR for *agrD* typing (related to Figure 4). PCR was conducted using the primers described in Table S1. Standard curves were prepared with the indicated copy numbers of the extracted genomic DNAs from *S. caprae* ATCC 35538 and JMUB145.

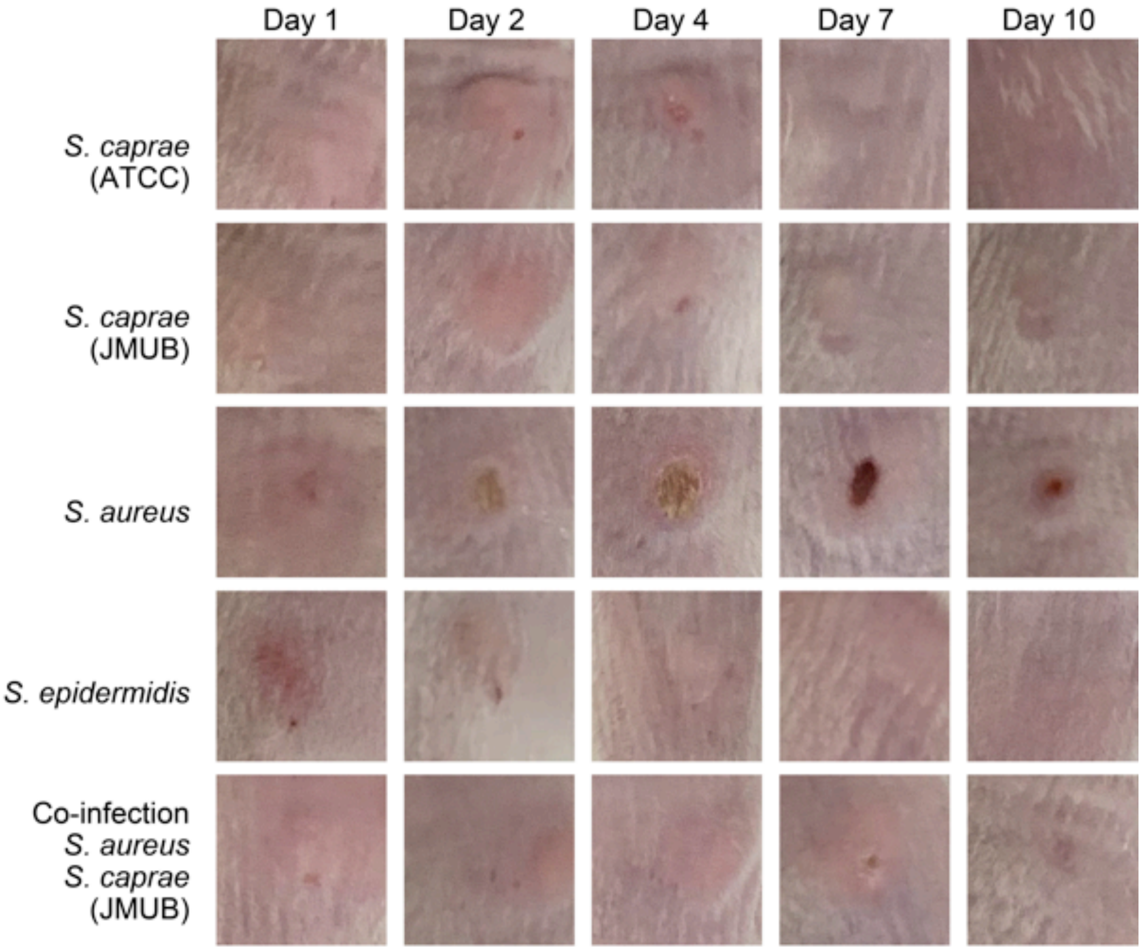

Figure S7. Representative images of the lesion after subcutaneous injection (related to Figure 6).

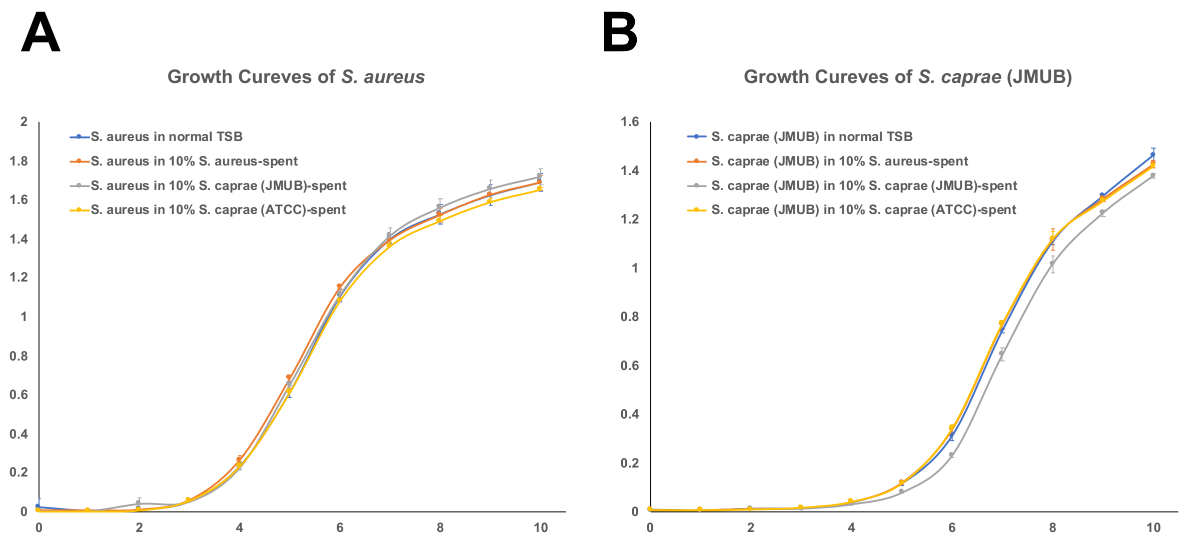

Figure S8. Growth curves of *S. aureus* and *S. caprae* in the presence of spent medium (related to Figure 7). (A) *S. aureus* N315 and (B) *S. caprae* JMUB145 were cultured in the absence (fresh TSB) or presence of spent medium (final 10% of spent medium in TSB).

Table S1. Primer sequences utilized in this study

Primers for Miseq Sequencing		Reference
SLST_1stPCR_fwd	5’-TCGTCGGCAGCGTCAGATGTGTATAAGAGACAGTGGCACGTAAACAAGTATC-3’[*1]	(Ederveen et al., 2019) (OG#421)
SLST_1stPCR_rvs	5’-GTCTCGTGGGCTCGGAGATGTGTATAAGAGACAGGACGACGTTTTGGTGGAC-3’ [*2]	(Ederveen et al., 2019) (OG#421)
Primers for quantitative PCR of *agrD* allele types		
agrD_YSTCSYYF_fwd	5’-GTGATCACTGCTGTTTTTGAA-3’	In this study
agrD_YSTCSYYF_rvs	5’-ATATTTCAAGTAGTTCTTTTGGTACTTCAG-3’	
agrD_YSTCSYYF_probe	5’-[FAM]TGGCTTTATAGCTGGTTATAGTACTTGTAGTTACT[MGBEQ]-3’	
agrD_YRTCNTYF_fwd	5’-GAGGATGTATGAAAATGATGCAAAT-3’	
agrD_YRTCNTYF_rvs	5’-GTTTCAAATAATTCTTTAGGTACTTCAGGT-3’	
agrD_YRTCNTYF_probe	5’-[FAM]GTTTTTTAGCTGGTTACAGAACATGTAATACT[MGBEQ]-3’	

[*1] The overhang adaptor sequence for MiSeq (CGTCGGCAGCGTCAGATGTGTATAAGAGACAG) was added to the OG#421 in Table S9 in the report.

[*2]The overhang adaptor sequence for MiSeq (GTCTCGTGGGCTCGGAGATGTGTATAAGAGACAG) was added to the OG#421 in Table S9 in the report.
